# Knowledge, attitude and practice of urban and rural households towards principles of nutrition in Iran: results of NUTRIKAP survey

**DOI:** 10.1186/s40200-014-0100-7

**Published:** 2014-12-04

**Authors:** Zeinab Ahadi, Ramin Heshmat, Maryam Sanaei, Gita Shafiee, Maryam Ghaderpanahi, Mohsen Rezaei Homami, Forouzan Salehi, Zahra Abdollahi, Bahar Azemati, Bagher Larijani

**Affiliations:** Chronic Diseases Research Center, Endocrinology and Metabolism Population Sciences Institute, Tehran University of Medical Sciences, Tehran, Iran; Endocrinology & Metabolism Research Center, Endocrinology and Metabolism Clinical Sciences Institute, Tehran University of Medical Sciences, Tehran, Iran; Rajaie Cardiovascular, Medical and, Research Center, Iran University of Medical Sciences, Tehran, Iran; Community Nutrition Department, Ministry of Health and Medical Education, Tehran, Iran; Department of Nutrition, School of Public Health, Loma Linda University, Loma Linda, CA USA

**Keywords:** Knowledge, Attitude, Practice, Nutrition, Urban, Rural

## Abstract

**Background:**

The aim of this study was to assess knowledge, attitude and practice of urban and rural households toward principles of nutrition in Iran.

**Methods:**

The study population was Iranian households who live in rural and urban areas in all provinces of the country. The sampling method at households’ level in each province was single stage cluster sampling with equal size clusters. The incumbent data was collected by a structured questionnaire and through the interview with the eligible subject in each household.

**Results:**

A total of 14,136 Iranian households were selected as total sample size, 9,149 urban households, and 4,987 rural households. Around 57.2% of urban and 49.5% of rural households was aware of food groups. Respectively in urban and rural households, about 35.1% and 39.7% had correct knowledge toward roles of food groups. Approximately 41.5% and 39.9% of households had accurate knowledge about reason of food eating in urban and rural areas, respectively. The results showed that 79.6% of them had favorable attitudes.

The most of the households consumed red meat and poultry weekly whereas fish was eaten rarely. Fruits, vegetables and dairy were consumed daily in the most of households. Sugar intake was daily in the most of households and cream and butter intake was weekly.

**Conclusion:**

The most of households had moderate knowledge and good attitudes. Practice of families about food consumption was good. The results of this study can be used for proper intervention for improving of health society.

## Introduction

Nutrition is an important factor in prevention of chronic diseases such as diabetes, obesity, cancer and cardiovascular diseases. The lifestyle transition with urbanization causing many problems, such as change of food intake pattern, decreasing physical activity, increasing salt and fat consumption that these factors result in increased prevalence of nutrition-related non-communicable diseases [1,2].

According to the Centers for Disease Control (CDC) “7 out of 10 deaths among Americans each year are from chronic diseases. Heart disease, cancer and stroke account for more than 50% of all deaths each year” [3]. In Iran, chronic diseases accounted for 70% of all deaths in 2002 [4]. So nutrition interventions are necessary for modifying the lifestyle and control the risk factors of these diseases. For that purpose, the factors affecting nutrition behavior should be identified. Level of nutrition knowledge and attitude are the important factors that influence the dietary practice. Nutrition knowledge may impress dietary practice directly or via nutrition attitude. Dietary behavior may further become pattern of food intake and impress one’s nutrient intake [5,6]. Thus understanding nutrition knowledge, attitude and behavior of people is the essential for the prevention of chronic diseases.

The study on knowledge, attitude and practice of Tehranian adults who participated in second phase of the Tehran lipid and glucose study about nutrition disclosed that for knowledge 26.5%, 52.7%, and 20.8%, for attitude, 27.6%, 48.9%, and 23.5% and for practice, 27.4%, 51.7%, and 20.9% of individuals had good, moderate and poor scores, respectively [7]. Yet, any study has not been carried out related to the Knowledge, attitude, and practice (KAP) among Iranian households, and comprehensive information is not available on KAP status. So this is necessary to obtain data about nutritional knowledge, attitude and behavior among people who live in Iran. The results of this study can be used for schematization and performance of effective interventions to improve nutritional status of society.

Thus the purpose of this study was to assess knowledge, attitude and practice of urban and rural households toward nutrition in Iran in 2011–2012.

## Materials and methods

This cross sectional study was performed to assess knowledge, Attitude and Practice (KAP) of urban and rural households toward principles of nutrition in 31 provinces, Iran. The methodology of this study was presented by details elsewhere [8].

### Subjects and sampling

The study population was Iranian households who live in rural and urban regions in 31 provinces of Iran. We considered mother or any member of the household who was above 15 yr old and responsible for cooking meals in the family as index case for data gathering. The sampling method at households’ level in each province was the single stage cluster sampling with equal size clusters. Regarding of design effect, total sample size was estimated 456 (57 clusters of 8 people in urban and rural areas of each province), so, in 31 provinces, 14,136 people participated in this study.

For finding first household of each cluster we used systematic random sampling method. We provided the cumulative list of households in each area based on 10-digit unique postal codes. The sampling interval was calculated (total number of households in each area divided by number of clusters in the area). The first household of the first cluster was identified by randomly selecting a number between 1 to a less than or equal to sampling interval. The first household of the second cluster was located by adding the sampling interval to the random number. For subsequent clusters (cluster 3, cluster 4, cluster 5, etc.), we identified the first household by adding the sampling interval to the running total of adding the sampling interval to the random number. The right side neighbors (clockwise direction) of the first household in each cluster were selected as the rest households of that cluster. Households were not from Iran and households were not present at the time of interview for three times excluded from the survey.

### Data collection

The incumbent data was collected by a structured questionnaire and through the interview with the eligible subject in each household. The validity and reliability of the questionnaire was evaluated in the pilot study. Cronbach’s alpha was calculated to assess the reliability and it was 0.79 [8]. The questionnaire developed for KAP had validity and reliability for assessing KAP toward nutrition among Iranian population. The collected data was regarding household’s knowledge in food groups, role of food groups and reason of food eating, attitude toward basic principles of nutrition and households’ practice or consumption food. The knowledge status was assessed by 7 items, and categories of response were “she/he knows” and “she/he does not know”. Attitude was measured by 10 items and categories of response were from 1 = I totally agree to 5 = I totally disagree. Practice was assessed by 10 items and answers ranged from “daily” to “never”.

### Statistical analyses

Date was analyzed by using the SPSS software package version 17 for windows (SPSS.Inc. Chicago, IL, USA). Data was analyzed based on sampling method (survey analysis) and 95% confidence intervals were calculated for presenting interval estimates. Estimations of each province were weighted proportional to the province population in its urban and rural areas to calculate national level estimations. We reported national level estimations in this article and provinces data were reported in detailed project report.

## Results

In this study, a total of 14,136 households were selected as total sample size, 9,149 urban households (64.9%), and 4,987 rural households (35.1%). Some households’ data were missed, so data analysis was based on households with complete information. More than 97% of respondents were women and about half of them (52%) were in the age range of 20–39 years (Table 1). The most frequent category of education reported by the respondents was high school diploma and below it (32%) in urban areas and was elementary (37.7%) in rural areas.

**Table 1:** **Respondents’ characteristics based on Region: the NUTRI-KAP survey**

	**Urban**	**Rural**	**Total**
**Sex**	N(%)	N(%)	N(%)
Male	231(2.5)	98(2.0)	329(2.3)
Female	8,889(97.5)	4,901(98.0)	13,790(97.67)
**Age**			
Less than 20 years old	187(2.0)	237(4.7)	424(3.0)
20-39 years old	4,508(49.5)	2,842(56.8)	7,350(52.1)
40-64 years old	3,722(40.8)	1,632(32.7)	5,354(37.9)
Above 65 years old	702(7.7)	288(5.8)	990(7.0)
**Education**			
Illiterate	1,643(18.0)	1,429(28.6)	3,072(21.8)
Elementary	2,222(24.4)	1,888(37.8)	4,110(29.1)
Intermediate	1,232(13.5)	651(13.0)	1,883(13.3)
High school	2,917(32)	851(17.0)	3,768(26.7)
Upper than diploma	1,103(12.1)	179(3.6)	1,282(9.1)
**SES**			
Weak	2,114(23.4)	2,555(51.3)	4,669(33.3)
Moderate	3,056(33.8)	1,614(32.4)	4,670(33.3)
Good	3,872(42.8)	811(16.3)	4,683(33.4)

Table 2 shows the Knowledge level of urban and rural households toward nutrition. Around 40-70% of urban households and 30-60% of rural households in various provinces were aware of food groups. The most of the respondents declared that they consumed food due to improvement their health and prevention of diseases. Twenty-nine percent (29%, CI 95%: 27.2-30.5) of urban and rural households knew butter and cream belong to the fat group.

**Table 2:** **Correct knowledge of households based on Region: the NUTRI-KAP survey**

		**Rural**	**Urban**	**Total**
**Recognition of food groups**		% (CI 95%)	% (CI 95%)	% (CI 95%)
Bread, Grains and Rice		59.2 (57.3- 61.0)	58.3 (55.5- 61.0)	58.9 (57.5- 60.4)
Meat and Legume		72.2 (70.2-74.0)	64.3 (62.1- 66.4)	69.8 (68.2- 71.4)
Milk and dairy group		55.5 (53.4- 57.7)	46.5 (44.6-48.5)	52.9 (51.0-54.7)
Fruits		44.7 (42.2-47.3)	35.1 (32.8-37.5)	41.9 (39.8- 43.9)
Vegetables		54.5 (52.2-56.7)	43.2 (40.8-45.7)	51.1 (49.2- 53.0)
**Roles of food groups**				
Bread, Grains and Rice		33.3 (31.5-35.2)	31.9 (29.4-34.5)	32.9 (31.5- 34.4)
Meat and Legume		47.8 (45.5-50.2)	31.6 (29.2-34.2)	43.0 (40.8- 45.2)
Milk and dairy group		76.6 (74.9-78.3)	63.6 (60.9- 66.2)	72.7 (71.0- 74.5)
Fruits	Vitamin intake	67.3 (65.0-69.5)	50.4 (48.1-52.6)	62.2 (59.9- 64.5)
	Mineral intake	16.7 (15.0-18.7)	13.3 (11.5-15.4)	15.7 (14.3- 17.2)
	Dietary fiber intake	11.8 (10.6-13.1)	6.3 (5.2-7.5)	10.2 (9.15- 11.3)
Vegetables	Vitamin intake	58.2 (55.9-60.5)	44.9 (42.5- 47.4)	54.2 (52.2-56.3)
	Mineral intake	25.0 (23.3-26.9)	61.0 (14.5- 17.8)	22.4 (21.0-23.8)
	Dietary fiber intake	21.4 (19.5- 23.4)	12.6 (10.9-14.5)	18.8 (17.3-20.3)
**Reason of food eating**				
Growth		26.3 (24.3- 28.4)	23.1 (21.1-25.1)	25.3 (23.8-26.8)
Energy intake		46.5 (44.5-48.5)	42.1 (40.1-43.9)	45.2 (43.6- 46.8)
Health and prevention of diseases		51.7 (49.7-54.1)	52.6 (50.3-55.0)	52.2 (50.6-53.7)

Table 3 shows the favorable attitudes of households toward basic principles of nutrition. The results revealed that 54-96% of families had favorable attitudes.

**Table 3:** **Favorable attitudes of households toward basic principles of nutrition based on Region: the NUTRI-KAP survey**

	**Urban**	**Rural**	**Total**
**Favorable attitudes**	% (CI 95%)	% (CI 95%)	% (CI 95%)
The importance of nutrition and diet in human health	97.2 (96.5-97.9)	96.3 (94.8-97.3)	97.0 (96.3- 97.5)
The necessity of same food intake in both sexes when a small amount of food is in the households basket	72.0 (70.1-73.8)	65.4 (62.6-68.1)	70.0 (68.3- 71.7)
The necessity of attention to the nutritional needs of children more than adults	91.8 (90.7-92.7)	89.5 (87.6-91)	91.1 (90.2- 91.9)
The observing fitness in girls at puberty with consume less food	56.6 (54.6-58.7)	50.8 (49.1- 52.4)	54.9 (53.2- 56.6)
The increase of nutritional needs of pregnant and lactating women compared to other women	94.9 (93.9-95.7)	95.6 (95.1- 96.1)	95.1 (94.4- 95.7)
The necessity of iron intake in children, even when the teeth become black	77.5 (75.5-79.4)	75.4 (73.0-77.8)	76.9 (75.3-78.4)
The necessity of regular use of iodized salt in cooking	93.4 (92.4-94.3)	95.5 (94.0- 96.6)	94.0 (93.2-94.8)
Wholemeal bread have more nutritional value than white bread	68.1 (66.3-69.9)	57.0 (54.9- 5.9.0)	64.8 (63.2-66.3)
The priority of traditional foods to fast food	93.6 (92.7-94.5)	93.1 (91.6- 94.3)	93.5 (92.6- 94.2)
The necessity of eating main meals in weight loss diet	60.2 (58.5-61.8)	55.2 (52.1- 58.3)	58.7 (57.2- 60.1)

Mostly, mother was the one who decided about grocery shopping in the most households in urban and rural households (65.8%, CI 95%: 63.6-68.0 and 58%, CI 95%: 55.1-60.8, respectively). Approximately 60-80% of households took breakfast every day and regularly. About 58 (CI 95%: 55.5-60.0) and 73% (CI 95%: 70.4-75.6) of urban and rural households cooked for every meal, respectively. In other cases they cooked maximum for two meals at once (Data not shown).

Table 4 shows the practice of households based on region. Findings indicated that the most of the households consumed red meat weekly in urban and rural households (72.9%, CI 95%: 71.5-74.3, and 66.9%, CI 95%: 64.6-69.1, respectively). The poultry consumption was weekly in the most of the households (urban households: 76.8%, CI 95%: 74.8-78.7, and rural households: 77.4%, CI 95%: 74.7-79.8). Fish was eaten weekly by the most of the urban families (45.1%, CI 95%: 43.5-46.8) whereas the most of the rural families (46.5%, CI 95%: 43.6-49.4) consumed it rarely. Respectively in urban and rural households, the frequency of weekly intake of egg was 65.8%( CI 95%: 64.2-67.3) and 62.5% (CI 95%: 60.7-64.2). Around 70% (CI 95%: 67.9-70.6) of households used legume weekly. 46.5% (CI 95%: 44.5-48.6) of urban families consumed sausage rarely while 55.3% (CI 95%: 52.2-58.4) of rural households never used it. Respectively in urban and rural households, the frequency of daily consumption of fruits was 78.3% (CI 95%: 76.9-79.6) and 60.5% (CI 95%: 57.3- 63.5), also for vegetables 65.3% (CI 95%: 63.4-67.2) and 49.2% (CI 95%: 45.9-52.6). The consumption frequency of dairy group was 86.2% (CI 95%: 84.8-87.5) and 79.9% (CI 95%: 77.2-82.3) in urban and rural families, respectively. Around 35% (CI 95%: 34.9-38.0) of households used butter and cream weekly. The frequency of daily sugar intake was 79.1% (CI 95%: 77.4-80.7) and 87.4% (CI 95%: 85.3-89.3) in urban and rural areas, respectively.

**Table 4:** **Practice of households based on Region: the NUTRI-KAP survey**

	**Urban**	**Rural**	**Total**
**Red meat**	% (CI 95%)	% (CI 95%)	% (CI 95%)
Daily	11.5(10.3-12.7)	9.0(7.4-10.8)	10.7(9.8-11.7)
Weekly	72.9(71.5-74.3)	66.9(64.6-69.1)	71.1(70.0-72.3)
Rarely	13.4(12.3-14.5)	21.0(19.2-23.0)	15.7(14.6-16.7)
Never	2.29(1.8-2.8)	3.1(2.2-4.3)	2.5(2.1-3.0)
**Poultry**			
Daily	13.8(12.4-15.3)	11.6(9.9-13.6)	13.2(12.0-14.4)
Weekly	76.8(74.8-78.7)	77.4(74.7-79.8)	77.0(75.3-78.6)
Rarely	8.5(7.6-9.5)	9.8(8.3-11.5)	8.9(8.1-9.8)
Never	0.8(0.6-1.2)	1.2(0.7-1.9)	0.9(0.7-1.2)
**Fish**			
Daily	1.7(1.2-2.3)	2.6(1.8-3.7)	2.0(1.6-2.4)
Weekly	45.1(43.5-46.8)	35.6(32.9-38.4)	42.3(40.5-44.1)
Rarely	42.7(41.2-44.2)	46.5(43.6-49.4)	43.8(42.4-45.3)
Never	10.5(9.3-11.9)	15.3(13.4-17.3)	11.9(10.8-13.2)
**Egg**			
Daily	23.4(21.9-25.0)	26.4(24.4-28.6)	24.3(23.1-25.6)
Weekly	65.79(64.2-67.3)	62.5(60.7-64.2)	64.8(63.8-66.0)
Rarely	9.2(8.0-10.7)	8.7(7.1-10.7)	9.1(8.1-10.1)
Never	1.5(1.2-2.0)	2.3(1.6-3.3)	1.8(1.4-2.2)
**Legume**			
Daily	22.5(20.9-24.3)	22.3(19.6-25.3)	22.5(21.0-24.0)
Weekly	68.7(66.9-70.4)	70.7(8.2-73.2)	69.3(67.9-70.6)
Rarely	7.5 (6.7-8.5)	5.3(4.1-6.9)	6.9(6.2-7.6)
Never	1.2(0.8-1.7)	1.6(1.3-2.0)	1.3(1.1-1.7)
**Fruits**			
Daily	78.3(76.9-79.6)	60.5(57.3-63.5)	73.0(71.0-74.8)
Weekly	17.8(16.6-19.0)	32.5(30.1-34.9)	22.2(20.6-23.8)
Rarely	2.9(2.4-3.5)	4.3(3.5-5.4)	3.3(2.8-3.9)
Never	1.0(0.8-1.4)	2.7(1.9-3.8)	1.5(1.2-2.0)
**Vegetables**			
Daily	65.3(63.4-67.2)	49.2(45.9-52.6)	60.5(58.5-62.5)
Weekly	29.2(27.5-30.9)	40.1(37.4-42.9)	32.4(30.9-34.0)
Rarely	4.19(3.4-5.1)	9.0(7.9-10.1)	5.6(4.9-6.5)
Never	1.3(1.0-1.8)	1.6(1.2-2.2)	1.4(1.1-1.7)
**Dairy**			
Daily	86.2(84.8-87.5)	79.9(77.2-82.3)	84.3(83.1-85.5)
Weekly	10.7(9.5-12.2)	16.6(14.4-19.1)	12.5(11.3-13.8)
Rarely	1.8(1.4-2.2)	2.2(1.7-2.9)	1.9(1.6-2.2)
Never	1.3(0.8-1.9)	1.3(0.8-2.2)	1.3(0.9-1.8)
**Butter and cream**			
Daily	27.8(26.4-29.3)	30.4(27.9-33.0)	28.6(27.3-29.9)
Weekly	36.9(35.2-38.8)	35.2(32.9-37.5)	36.4(34.9-38.0)
Rarely	22.4(20.6-24.3)	25.0(22.9-27.3)	23.2(21.6-24.8)
Never	12.8(11.4-14.3)	9.4(7.7-11.5)	11.8(10.6-13.1)
**Sugar**			
Daily	79.1(77.4-80.7)	87.4(85.3-89.3)	81.6(80.0-83.1)
Weekly	7.8(6.6-9.1)	5.3(4.5-6.4)	7.1(6.2-8.1)
Rarely	8.7(7.8-9.8)	4.0(3.3-5.0)	7.3(6.6-8.2)
Never	4.4(3.6-5.2)	3.2(2.3-4.3)	4.0(3.3-4.8)

Source of dietary and nutrition information based on region has shown in Table 5. In the most of urban and rural households, the major source of nutrition information was TV programs. About 46% (CI 95%: 44.0-48.0) and 38% (CI 95%: 35.4-41.2) of urban and rural families considered TV and health units as preferred sources of nutrition education, respectively (Data not shown). Around 27% (CI 95%: 26.1-29.0) and 34% (CI 95%: 32.0-26.3) of urban and rural households were willing to learn about the principles of proper diet and the correct method of cooking and maintenance of foods, respectively (Data not shown).

**Table 5:** **Sources of dietary and nutrition information based on Region: the NUTRI-KAP survey**

	**Urban**	**Rural**	**Total**
**Sources**	% (CI 95%)	% (CI 95%)	% (CI 95%)
Health units	11.1 (9.7-12.5)	31.1 (28.0-34.3)	17.0(14.7-19.2)
TV programs	54.8 (52.6-56.9)	49.1 (45.7- 52.4)	53.2 (51.4-54.8)
Newspaper and magazines	10.3 (9.1-11.6)	5.3 (4.0- 6.9)	8.8 (7.8- 10.0)
Friends and neighbors	1.8 (1.3-2.4)	0.9 (0.5- 1.5)	1.5 (1.1- 2.0)
Family member	2.5 (2–3.3.0)	2.0 (1.5- 2.6)	2.2 (1.9-2.9)
Experiencing women in families	1.5 (1.2- 2.0)	1.4 (1.1- 2.2)	1.5 (1.2- 1.9)
Medical practitioner	5.6 (4.6- 6.9)	4.6 (3.6- 5.8)	5.3 (4.5-6.3)
Dietitian	5.6 (4.6-6.9)	1.1 (0.7-1.6)	4.3 (3.6-5.1)
Other	6.8 (6–7.7.0)	4.5 (3.5- 5.8)	6.2 (5.5- 6.9)

## Discussion

The aim of this study was to assess knowledge, attitude and practice of urban and rural households toward principles of nutrition in Iran. In our study, the most of respondents were woman. This result showed that mothers play important role in nutrition of family. As a result, mothers’ nutrition knowledge is key influence on quality of households’ diets.

According to the results of this study, the knowledge level of urban and rural households was acceptable about recognition of food groups. The results showed that the most of the households were knowledgeable about the role of milk and dairy group in growth and strength of bones and teeth whereas had less knowledge about the role of grains group (generation of energy and power for doing work) and meat, legume and egg group (providing of protein for growth and evolution). The most of the households expressed that the importance of fruits and vegetables intake was provided of vitamins, mineral and dietary fiber, respectively. They indicated that they consumed food due to improvement their health and prevention of diseases and then energy intake and in order to growth. The knowledge level of households about dairy group and the importance of fruits and vegetables in providing vitamins were good and acceptable but about bread, grain and meat group was weak as well as the importance of fruits/vegetables in providing mineral and dietary fiber. So they needed to be educated about these fields.

Farivar et al. assessed knowledge, attitude and practice of urban households toward nutrition in Boushehr, Golestan and Sistan & Balouchestan provinces in 2004. The study results showed that the correct knowledge level of these provinces households about recognition of food groups were similar to our study, while the knowledge level of them toward the role of food groups were different from present study. They reported that the most reason of food eating was health and prevention of diseases that were similar to results of our study [9]. In another studies of these provinces, KAP of them about iron deficiency anemia, osteoporosis and osteomalacia were determined that both of them were not acceptable [10], that method of our study was similar theirs with wider population and representative of the entire country [11]. Lainez et al.’s study estimated knowledge and attitude of the Canary Island population toward eating in relation to health. 46.7% of participants considered their knowledge of food and nutrition to be sufficient [12]. Ostadrahimi et al. investigated the effect of education on nutrition knowledge, attitude and practice of employed women in Tabriz University. The results indicated that 82.5, 16.5 and 0.9% of them had good, moderate and weak knowledge, respectively. After the education, their knowledge did not improve significantly [13]. Although the survey on Malaysian elderly showed that 39 and 20% of them had desirable and poor knowledge scores [14]. Mirmiran et al. reported that 26.5, 52.7 and 20.8% of urban Tehranian adults who participated in second phase of the Tehran Lipid and Glucose Study had good, moderate and poor knowledge scores, respectively [7].

The results of NUTRIKAP study showed that urban and rural households expressed favorable attitudes toward nutrition. The results revealed that more than of 50% of families had positive attitude, although Farivar et al. reported that less than 60% of urban households had favorable attitudes [9]. Lin et al. affirm that there are significantly positive correlations among nutrition knowledge, attitude and practice; and attitude has stronger association with practice than knowledge does [15].

In the present study, the frequency of poultry’s consumption was more than red meat and fish. Around 43 and 47% of urban and rural households consumed fish less than one time in week, 10 and 15% of them never eat fish, respectively. The American Heart Association’s dietary guidelines recommend that healthy adults intake at least two servings of fish per week [16]. The consumption of fish may protect against cardiovascular disease and stroke because of proteins, vitamins, minerals, and especially omega-3 polyunsaturated fatty acids (PUFAs) [17]. Thus, we should identify the factors that lead to decreased fish consumption for improvement of fish intake pattern in households.

Fruits and vegetables are rich sources of antioxidant that these compounds reduce the risk of major chronic diseases [18]. Regular consumption of fruits and vegetables is associated with reduce risk of cardiovascular disease, cancer, Alzheimer disease and stroke [19]. In this study, more than 60% of households’ fruits and vegetables intake was daily except rural households that 49% of them consumed vegetables every day. The frequency of fruits and vegetables intake of Golestan and Sistan & Balouchestan households in Farivar’s study was similar to households’ intake in our study [9].

In the current study, nearly 80% of household consumed sugar every day and the most of the households never eat chips and sausage. Sugar intake is associated with dental caries, the increased rate of obesity and decreased fruits and vegetables intake [20-22]. Therefore, the decreased sugar intake is necessary for prevention of its outcomes.

According to findings of this study, the main sources of nutrition information for urban and rural households included TV, health units, newspaper and magazines. As regards TV was favorite source of nutrition information, how to better use TV as a medium for progress of knowledge, attitude and practice and as a result, to promote of dietary pattern and public health.

## Conclusion

The results of our study declared that nutrition knowledge of mother determined the dietary pattern of households in Iran. Nutritional intervention such as nutrition education programmes might be necessary for promotion of health and nutrition status in the future.

## References

[1] Ghassemi H, Harrison G, Mohammad K (2002). An accelerated nutrition transition in Iran. Public Health Nutr.

[2] Nissinen A, Berrios X, Puska P (2001). Community-based noncommunicable disease interventions: lessons from developed countries for developing ones. Bull World Health Org.

[3] **Chronic disease and health promotion.** [http://www.cdc.gov/chronicdisease/overview/index.htm]

[4] **The impact of chronic disease in the Islamic Republic of Iran.** [http://www.who.int/chp/chronic_disease_report/media/impact/en/].

[5] Grotkowski ML, Sims LS (1978). Nutritional knowledge, attitudes, and dietary practices of the elderly. J Am Diet Assoc.

[6] McIntosh W, Kubena K, Walker J, Smith D, Landmann W (1990). The relationship between beliefs about nutrition and dietary practices of the elderly. J Am Diet Assoc.

[7] Mirmiran P, Mohammadi-Nasrabadi F, Omidvar N, Hosseini-Esfahani F, Hamayeli-Mehrabani H, Mehrabi Y, Azizi F (2010). Nutritional knowledge, attitude and practice of Tehranian adults and their relation to serum lipid and lipoproteins: Tehran lipid and glucose study. Ann Nutr Metab.

[8] Azemati B, Heshmat R, Sanaei M, Salehi F, Sadeghi F, Ghaderpanahi M, Mirarefin M, Abdollahi Z, Hemami MR, Larijani B (2013). Nutritional knowledge, attitude and practice of Iranian households and primary health care staff: NUTRIKAP Survey. J Diabetes Metab Disord.

[9] Farivar F, Heshmat R, Aemati B, Abbaszadeh Ahranjani S, Keshtkar A, Sheykh-ol-Eslam R, Nadim AH (2009). Knowlwdge, Attutide and practice of urban households toward principles of nutrition. Iran J Epidemiol.

[10] Heshmat R, Azemati B, Keshtkar A, Salehi F, Abdollahi Z, Kolahdouz F, PourAram H, Farivar F, Bagheri M, Sheykh-ol-Eslam R, Nadim A (2009). Comparison of Knowledge, Attitude and Practice of Urban and Rural Households toward Iron Deficiency Anemia in three Provinces of Iran. Iran J Epidemiol.

[11] Heshmat R, Keshtkar A, Sheykh-ol-Eslam R, Nadim A, Bagheri M (2005). Knowledge, Attitude and Practice of Urban Households towards nutrition and micronutrients(NUT-KAP) in 3 provinces of Iran. Iran J Epidemiol.

[12] Lainez P, Navarro Rodriguez MC, Male Gil ML, Serra Majem L (2000). Knowledge, opinions and attitudes of the Canarian Islands population towards nutrition. Arch Latinoam Nutr.

[13] Ostadrahimi A, Safaeian Z, Modaresi P, Mahdavi R (2009). The effect of Education on knowledge, Attitude and Practice of Employed Women in Tabriz University. Med J Tabriz Univ.

[14] Pon L, Noor-Aini M, Ong F, Adeeb N, Seri S, Shamsuddin K, Mohamed A, Hapizah N, Mokhtar A, Wan H (2006). Diet, nutritional knowledge and health status of urban middle-aged Malaysian women. Asia Pac J Clin Nutr.

[15] Lin W, Hang CM, Yang HC, Hung MH (2011). 2005–2008 Nutrition and Health Survey in Taiwan: the nutrition knowledge, attitude and behavior of 19–64 year old adults. Asia Pac J Clin Nutr.

[16] Kris-Etherton PM, Harris WS, Appel LJ (2002). Fish consumption, fish oil, omega-3 fatty acids, and cardiovascular disease. Circulation.

[17] Domingo JL (2007). Omega-3 fatty acids and the benefits of fish consumption: is all that glitters gold?. Environ Int.

[18] Southon S (2000). Increased fruit and vegetable consumption within the EU: potential health benefits. Food Res Int.

[19] Liu RH (2003). Health benefits of fruit and vegetables are from additive and synergistic combinations of phytochemicals. Am J Clin Nutr.

[20] Lewis C, Park Y, Dexter PB, Yetley E (1992). Nutrient intakes and body weights of persons consuming high and moderate levels of added sugars. J Am Diet Assoc.

[21] Gibson SA (2008). Consumption and sources of sugars in the diets of British schoolchildren: are high‐sugar diets nutritionally inferior?. J Hum Nutr Diet.

[22] Overby N, Lillegaard ITL, Johansson L, Andersen LF (2004). High intake of added sugar among Norwegian children and adolescents. Public Health Nutr-Cab Int.

